# Mechanical effects of left ventricular midwall fibrosis in non-ischemic cardiomyopathy

**DOI:** 10.1186/s12968-015-0221-2

**Published:** 2016-01-05

**Authors:** Robin J. Taylor, Fraz Umar, Erica L. S. Lin, Amar Ahmed, William E. Moody, Wojciech Mazur, Berthold Stegemann, Jonathan N. Townend, Richard P. Steeds, Francisco Leyva

**Affiliations:** 1 Centre for Cardiovascular Sciences, University of Birmingham, Birmingham, UK; 2 Department of Cardiology, The Queen Elizabeth Hospital Birmingham, Mindelsohn Way, Edgbaston, Birmingham, B15 2WB UK; 3 The Christ Hospital Heart and Vascular Center, Cincinnati, OH USA; 4 Bakken Research Centre, Medtronic Inc, Maastricht, The Netherlands; 5 Aston Medical Research Institute, Aston Medical School, University of Aston, Birmingham, UK

**Keywords:** Heart failure, Non ischemic dilated cardiomyopathy, Mid-wall fibrosis, Feature-tracking, Cardiovascular magnetic resonance, Torsion, Myocardial deformation

## Abstract

**Background:**

Left ventricular (LV) mid-wall fibrosis (MWF), which occurs in about a quarter of patients with non-ischemic cardiomyopathy (NICM), is associated with high risk of pump failure. The mid LV wall is the site of circumferential myocardial fibers. We sought to determine the effect of MWF on LV myocardial mechanics.

**Methods:**

Patients with NICM (*n* = 116; age: 62.8 ± 13.2 years; 67 % male) underwent late gadolinium enhancement cardiovascular magnetic resonance (CMR) and were categorized according to the presence (+) or absence (−) of MWF. Feature tracking (FT) CMR was used to assess myocardial deformation.

**Results:**

Despite a similar LVEF (24.3 vs 27.5 %, p = 0.20), patients with MWF (32 [24 %]) had lower global circumferential strain (Ɛ_cc_: −6.6 % vs −9.4 %, *P* = 0.004), but similar longitudinal (Ɛ_ll_: −7.6 % vs. −9.4 %, *p* = 0.053) and radial (Ɛ_rr_: 14.6 % vs. 17.8 % *p* = 0.18) strain. Compared with − MWF, + MWF was associated with reduced LV systolic, circumferential strain rate (−0.38 ± 0.1 vs −0.56 ± 0.3 s^−1^, *p* = 0.005) and peak LV twist (4.65 vs. 6.31°, *p* = 0.004), as well as rigid LV body rotation (64 % vs 28 %, *P* <0.001). In addition, +MWF was associated with reduced LV diastolic strain rates (DSR_cc_: 0.34 vs. 0.46 s^−1^; DSR_ll_: 0.38 vs. 0.50s^−1^; DSR_rr_: −0.55 vs. −0.75 s^−1^; all *p* <0.05).

**Conclusions:**

MWF is associated with reduced LV global circumferential strain, strain rate and torsion. In addition, MWF is associated with rigid LV body rotation and reduced diastolic strain rates. These systolic and diastolic disturbances may be related to the increased risk of pump failure observed in patients with NICM and MWF.

## Background

Non-ischemic cardiomyopathy (NICM) is a common cause of heart failure [1]. The NICM phenotype ranges from patients who remain largely asymptomatic to those who succumb to multiple hospitalizations and premature death. In a study of 603 patients with idiopathic dilated cardiomyopathy followed up over 9 years, Castelli et al. found that 45 % died or underwent cardiac transplantation [2].

Left ventricular mid-wall fibrosis (MWF) was first described as an autopsy finding in 1991 [3]. Clinical studies using late-gadolinium cardiovascular magnetic resonance (LGE-CMR) have subsequently shown that in patients with NIDCM, MWF is associated with an increased risk of heart failure hospitalizations, ventricular arrhythmias and cardiac death [4–8]. Patients with NICM and MWF are also less responsive to pharmacologic therapy [9] and cardiac resynchronization therapy [10]. Whilst the evidence linking MWF and poor patient outcomes is compelling [4–11], the mechanism remains unexplored.

The left ventricle (LV) twists in systole and untwists, or recoils, in diastole. In systole, the LV base rotates clockwise and the apex rotates counter-clockwise. This wringing motion is effected by the helical arrangement of myocardial fibers, which run in a left-handed direction in the subepicardium and in a right-handed direction in the subendocardium . Contraction of subepicardial myocardial fibers cause the base to rotate clockwise and the apex to rotate in counterclockwise [12]. Because the radius of rotation of the subepicardium is greater than that of the subendocardium, the former provides a greater torque. Consequently, the LV gets smaller in systole and LV ejection occurs [12]. Circumferential fibers, which run in the mid-myocardium, are crucial to this process. During ejection, they shorten simultaneously with oblique fibers in the right- and left-handed helices. In effect, circumferential fibers provide a horizontal counterforce throughout ejection [13].

We hypothesized that injury to mid-myocardial, circumferential myocardial fibers [14], as might be expected from MWF, leads to impairment of LV circumferential contraction and relaxation and therefore, to disturbances in LV twist and torsion. In this study, we have used feature-tracking CMR (FT-CMR) [15] to explore the mechanical effects of MWF in patients with NICM.

## Methods

### Patients

Patients with NICM were recruited through CMR units in two centers (Good Hope Hospital and Queen Elizabeth Hospital, Birmingham, United Kingdom). The initial diagnosis of cardiomyopathy was made on the basis of clinical history, echocardiographic evidence of LV systolic impairment and absence of coronary artery disease on invasive coronary angiography. The diagnosis of NICM was also made on the basis of LGE-CMR [4]. Mid-wall LGE was assessed visually and only deemed to be present if a crescentic or circumferential area of mid-wall signal enhancement (2 SD above the mean intensity of remote myocardium in the same slice [16]), surrounded by non-enhanced epicardial and endocardial myocardium was evident. Patients with scars in a sub-endocardial or transmural distribution following coronary artery territories were regarded as ischemic in etiology [4] and excluded. Those with epicardial, transmural or patchy fibrosis suggestive of other etiologies were also excluded. It is routine clinical practice at the two recruiting dedicated heart failure units to perform CMR as part of the diagnostic work-up. Accordingly, all patients underwent CMR at the time of the diagnosis. All Participants gave written informed consent, and the study protocol conformed to the Declaration of Helsinki and was approved by the National Research Ethics Service.

### CMR

This was undertaken using 1.5 Tesla Magnetom Avanto (Siemens, Erlangen, Germany) or Signa (GE Healthcare Worldwide, Slough, England) scanners and a phased-array cardiac coil. A horizontal long-axis image and a short-axis LV stack from the atrioventricular ring to the LV apex were acquired using a steady state in free precession (SSFP) sequence (repetition time of 3.2 ms; echo time of 1.7 ms; flip angle of 60°; sequential 7 mm slices with a 3 mm interslice gap). There were 25 phases per cardiac cycle resulting in a mean temporal resolution of 40 ms.

For scar imaging, horizontal and vertical long-axis as well as short-axis slices identical to the LV stack were acquired using a segmented inversion-recovery technique 10 min after the intravenous administration of gadolinium-diethylenetriamine pentaacetic acid (0.1 mmol/kg). Inversion times were adjusted to null normal myocardium (260 to 400 ms). To exclude artefact, we required the typical scar pattern to be visible in the short-axis and long-axis acquisitions, in two different phase encoded directions. Patients were dichotomized according to the presence or absence of MWF, assessed visually by an experienced observer (F.L.), who was blinded to other study data.

Feature tracking CMR (Tomtec Imaging Systems, Munich, Germany) was undertaken as previously described. It has been validated against myocardial tagging for the assessment of myocardial mechanics [15, 17]. We have previously shown that both circumferential- and longitudinal-based variables have an excellent intra- and inter-observer variability [18]. Global peak systolic circumferential (Ɛ_cc_) and radial (Ɛ_rr_) strain, strain rates (SSR_cc_ and SSR_rr_) and diastolic strain rates (DSR_cc_ and DSR_rr_) were assessed using FT-CMR of the mid-cavity LV short-axis cine. Longitudinal strains (Ɛ_ll_, SSR_ll_ and DSR_ll_) were assessed using the horizontal long axis cine. Only the SSFP sequences were uploaded onto the FT-CMR software, ensuring that the operator (R.T.) was blinded to MWF status. In addition, MWF status was decided by an investigator (F.L.) who was blinded to the findings of FT-CMR.

**Fig. 1:**
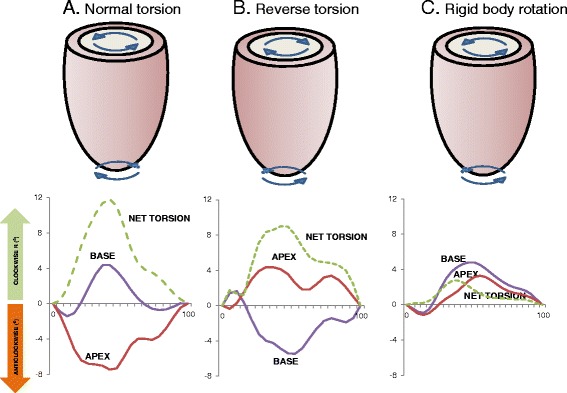
Rotational mechanics in NICM. Diagrammatic representation of torsional and rotational patterns identified using feature-tracking cardiovascular magnetic resonance. In the bottom tiles, the time in the cardiac cycle, expressed as a percentage of the R-R interval on the ECG, is shown in the x axes. Rotation at the base and apex of the LV as well as net torsion (the instantaneous difference between apical and basal rotation) is shown on the y axis (*in degrees*) **a** shows a preserved torsional pattern from a patient with non-ischemic dilated cardiomyopathy without MWF with predominantly anticlockwise rotation at the apex and clockwise rotation at the base. **b** shows reverse torsion, where the direction of both apical and basal rotation is reversed. **c** shows rigid body rotation in a patient with NICM and MWF. The apex and base both twist in the same direction so that the heart rotates as one solid body with minimal net torsion

Peak systolic rotation was measured using the basal and apical short axis cines. In health, peak systolic rotation, as viewed from the apex, is typically clockwise (+) at the base, and anti-clockwise (−) at the apex. Peak systolic rotation was calculated in degrees and expressed as both the maximum extent of rotation in the anticipated direction (i.e., if systolic rotation at the apex was solely in a clockwise direction this would equate to 0°) and the total magnitude of rotation (regardless of direction). Torsional parameters are derived from the peak instantaneous net difference in apical and basal rotation. LV twist was defined as (Φ _apex_ - Φ _base_), twist per unit length (Φ _apex_ - Φ _base_/D), and LV torsion (circumferential-longitudinal shear angle) as (Φ _apex_ - Φ _base_)(ρ _apex_ - ρ _base)_ / 2D (where Φ = the rotation angle; ρ = epicardial radius, and; D = base-to-apex distance) in accordance with agreed methodologies [19]. Systolic torsion was classified as either: a) normal torsion, in which there is predominantly anticlockwise rotation of the apex and clockwise rotation of the base;b) rigid body rotation: both the apex and base rotating in the same direction; and c) reverse torsion: predominantly clockwise rotation of the apex and anti-clockwise rotation of the base (Fig. 1).

### Statistical analysis

Categorical variables were expressed as a percentage and continuous variables as mean ± standard deviation (SD). Normality was tested using the Shapiro-Wilk test. Comparisons between variables were made with Fisher’s exact test for categorical variables and independent samples t-tests for continuous variables, after adjustment by the Welch-Satterthwaite method where Levene’s test showed unequal variance between groups. A *p* value of <0.05 was considered statistically significant for all tests. Statistical analyses were performed using SPSS v21.0. (SPSS Inc. Chicago, Illinois).

## Results

The characteristics of the study group are shown in Table 1. Amongst the entire cohort, 32/116 patients (28 %) had MWF. Patients were of similar age (63.8 vs. 62.3 years, *p* = 0.29), but more patients with MWF were men (84 % vs. 61 %, *p* = 0.02). There were no differences in NHYA class, atrial rhythm, QRS duration, LVEF, co-morbidities, pharmacological therapy for heart failure.

**Table 1:** Baseline characteristics

	No MWF	MWF	P
N	84	32	
Age, yrs	62.3 ± 13.7	63.8 ± 11.9	0.29
Male, n (%)	51 (61)	27 (84)	0.02
Height, m	1.68 ± 0.09	1.74 ± 0.09	0.02
Weight, Kg	83.4 ± 18.6	83.3 ± 12.6	0.97
NYHA class			0.20
I	4 (5)	3 (9)	
II	15 (18)	8 (25)	
III	47 (56)	11 (34)	
IV	9 (11)	5 (16)	
Unknown	9 (11)	5 (16)	
Diabetes mellitus, n (%)	13 (16)	7 (24)	0.42
Hypertension, n (%)	18 (22)	5 (17)	0.61
Atrial fibrillation, n (%)	15 (18)	8 (24)	0.44
Medication, n (%)			
Loop diuretics	62 (81)	26 (89)	0.47
ACE-I or ARB	77 (97)	27 (90)	0.31
Beta-blockers	51 (65)	20 (66)	1.00
Aldosterone antagonists	36 (46)	10 (35)	0.29
Systolic blood pressure, mmHg	124.3 ± 20.5	119.6 ± 23.1	0.38
Diastolic blood pressure, mmHg	71.5 ± 11.9	71.7 ± 13.8	0.96
QRS duration (ms)	144 (28)	149 (32)	0.48

*ACE-I* angiotensin-converting enzyme inhibitors, *ARB* angiotensin receptor blockers

### Systolic deformation

**Fig. 2:**
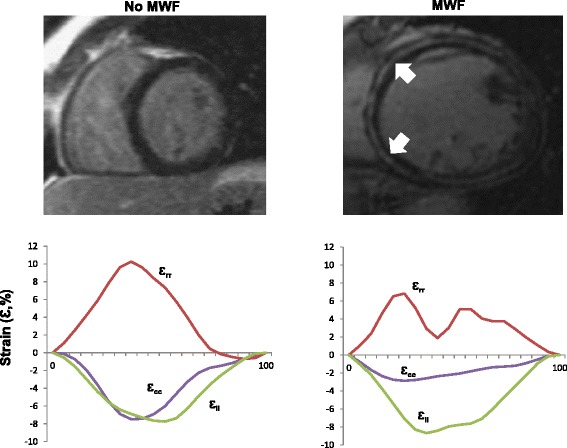
Feature-tracking CMR. Short-axis, late gadolinium enhancement views of patients with idiopathic dilated cardiomyopathy, without and with mid-wall fibrosis (MWF, *white arrows*). The bottom tiles show plots of global circumferential strain (Ɛ_cc_, *purple*), global radial strain (Ɛ_rr_, *red*) and global longitudinal strain (Ɛ_ll_, *green*) over a cardiac cycle. Note the marked reduction in Ɛ_cc_ in the patient with MWF

**Fig. 3:**
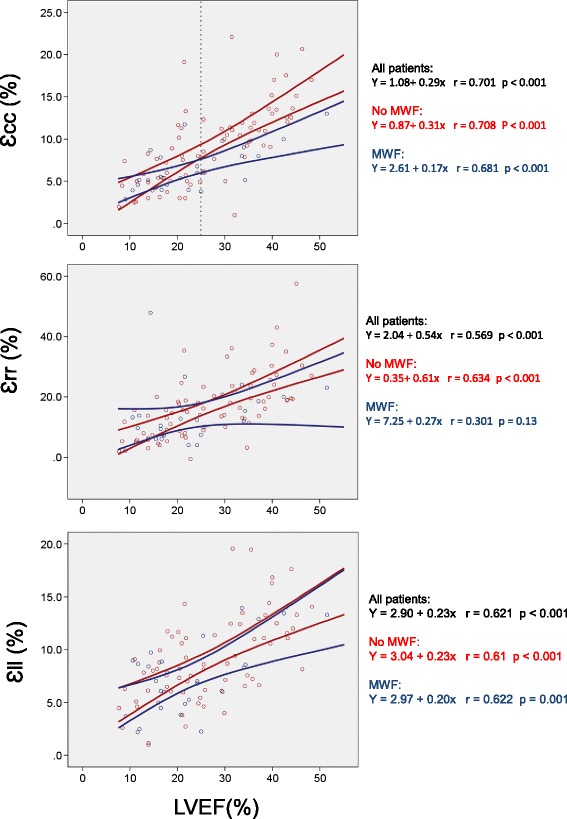
Relationship between LVEF and myocardial strain. Scattergrams for each of the Lagrangian strains plotted against LVEF. Cases are classified according to presence (*blue circles*) or absence (*red circles*) of mid-wall fibrosis (MWF). The lines correspond to the 95 % confidence intervals for strain. The top scattergram demonstrates that above an LVEF of 25 % (*dashed reference line*) MWF alters the relationship between Ɛ_cc_ and LVEF: patients with MWF have lower Ɛ_cc_ than those with similar LVEF but without MWF

As shown in Table 2, patients with MWF had a lower, global circumferential strain (Ɛ_cc_: −6.6 % vs −9.4 %, *P* = 0.004), but similar longitudinal (Ɛ_ll_: −7.6 % vs. −9.4 %, p −0.053) and radial (Ɛ_rr_: 14.6 % vs. 17.8 % *p* = 0.18) strain. Systolic strain rate was reduced in the circumferential direction (SSR_cc_: −0.38 s^−1^ vs. −0.56 s-1, *p* = 0.005), but not in radial or longitudinal directions. Figure 2 shows typical examples. As shown in Fig. 3, Ɛ_cc_ (r = 0.70), Ɛ_rr_ (0.57, *p* <0.001 and Ɛ_ll_ (r = 0.62, *p* <0.001) correlated positively with LVEF. In the case of Ɛ_cc_, the slope of the regression line was 0.17 in the + MWF group and 0.31 in the -MWF group, indicating that Ɛ_cc_ is lower in the + MWF group than in the -MWF at a given LVEF.

**Table 2:** Mechanical variables in patients with or without MWF

	No MWF	MWF	P
LV dimensions			
LVEDV, mL	222 ± 80	277 ± 79	0.002
LVESV, mL	166 ± 79	214 ± 83	0.007
LV mass, g	137.6 ± 46.6	155.5 ± 71.1	0.052
Systolic deformation			
LVEF, %	27.5 ± 10.8	24.3 ± 12.9	0.20
Ɛcc (%)	−9.4 ± 4.76	−6.6 (2.57	0.004
SSR_cc_ (s^−1^)	−0.56 ± 0.25	−0.38 (0.12	0.005
Ɛ_rr_ (%)	17.8 ± 11.0	14.6 ± 10.1	0.18
SSR_rr_ (s^−1^)	0.84 ± 0.37	0.74 ± 0.40	0.31
Ɛ_ll_ (%)	−9.4 ± 4.35	−7.6 ± 3.34	0.053
SSR_ll_ (s^−1^)	0.56 ± 0.20	−0.49 ± 0.18	0.13
Diastolic deformation			
DSR_cc_ (s^−1^)	0.46 ± 0.19	0.34 ± 0.11	0.010
DSR_rr_ (s^−1^)	−0.75 ± 0.35	−0.55 ± 0.44	0.038
DSR_ll_ (s^−1^)	0.50 ± 0.20	0.38 ± 0.14	0.006
Systolic torsion			
Basal systolic rotation (°)			
Net Clockwise	3.40 ± 3.00	3.00 ± 2.23	0.513
Magnitude	4.63 ± 2.64	3.67 ± 1.97	0.082
Basal rotation rate (° s^−1^)	31.3 ± 14.5	22.1 ± 8.2	0.002
Apical systolic rotation (°)			
Net anti-clockwise	−3.50 ± 3.28	−1.99 ± 1.97	0.024
Magnitude	5.18 ± 3.15	3.52 ± 2.45	0.013
Apical rotation rate (° s^−1^)	−38.9 ± 21.8	−26.1 ± 15.8	0.005
Average basal/apical rotation (°)	9.81 ± 4.48	7.20 ± 3.44	0.002
LV twist (°)	6.31 ± 3.30	4.65 ± 2.18	0.004
LV twist per unit length (°/cm)	1.34 ± 0.76	0.94 ± 0.55	0.005
Torsional shear angle	0.83 ± 0.06	0.52 ± 0.07	0.008
LV twist rate (° s^−1^)	48.4 ± 23.1	36.1 ± 17.1	0.01
Torsional pattern			<0.001
Normal torsion, n (%)	39 ± 46	10 ± 32	
Rigid body rotation, n (%)	23 ± 28	21 ± 64	
Reverse torsion, n (%)	22 ± 26	1 ± 4	
Diastolic torsion			
Basal rotation rate (° s^−1^)	−34.1 ± 14.8	−28.0 ± 11.8	0.053
Apical rotation rate (° s^−1^)	38.3 ± 20.1	24.9 ± 13.1	0.001
LV untwist rate (° s^−1^)	44.5 ± 21.0	30.5 ± 14.9	<0.001

Variables are expressed as mean ± SD

*MWF* mid-wall fibrosis, *SSR* systolic strain rate, *DSR* diastolic strain rate, Ɛ strain

### Diastolic deformation

In patients with MWF, diastolic strains rates were lower in all three directions in patients with MWF (DSR_cc_: 0.34 vs 0.46 s^−1^, *p* = 0.01; DSR_rr_: −0.55 vs −0.75 s^−1^, *p* = 0.04; DSR_ll_: 0.38 vs 0.50 s^−1^, *p* = 0.006).

### Torsional mechanics

Whilst basal rotation was unaffected by MWF (net clockwise: 3.00° vs. 3.30, *p* = 0.51; total magnitude: 3.67° vs. 4.63°, *p* = 0.08), the rate of basal rotation was reduced (22.1° s^−1^ vs 31.3° s^−1^, *p* = 0.002). In patients with MWF, apical rotation was also reduced in terms of both the total magnitude (3.52° vs 5.18°, *p* = 0.013) and the net anti-clockwise rotation (−1.99° vs. −3.50°, *p* = 0.024). The rate of apical rotation was lower in patients with MWF (−26.1° s^−1^ vs −38.9° s^−1^, *p* = 0.005). This reduction in the magnitude of apical rotation was associated with a reduction in LV twist (peak LV twist : 4.65° vs. 6.31°, *p* = 0.004; LV twist per unit length: 0.94°/cm vs.1.34°/cm, *p* = 0.005; torsional shear angle: 0.52 vs. 0.83, *p* = 0.008). The rate of LV twist (36.1° s^−1^ vs. 48.4° s^−1^, *P* = 0.001) and untwist (30.5° s^−1^ vs. 44.5° s^−1^, *P* <0.001) was also reduced in patients with MWF. A normal torsion pattern, in which there is predominantly anti-clockwise rotation of the apex and clockwise rotation of the base, was observed more frequently in patients without MWF (32 vs 46 %). Rigid LV body rotation was more frequently observed in patients with MWF (64 vs 28 %, *p* <0.001).

## Discussion

In this study, we have shown that in patients with NICM, MWF is associated with a selective impairment of circumferential LV myocardial strain. In addition, MWF is associated with impaired apical rotation and a reduction in rotation rate, from base to apex. MWF is also associated with impaired diastolic function, reflected in reductions in untwist in all directions, from base to apex. Together, these findings are consistent with the notion that, by affecting predominantly circumferential myocardial fibers, MWF leads to disturbances in myocardial contraction and diastolic function. The result is a 'stiff' LV, which is less able to twist to an applied torque (rotation) and more likely to move as a solid body. These disturbances may be related to the known associations of MWF with reduced pump function, heart failure hospitalizations and a poor response to medical and device therapy [4–11].

### Systole

During ejection, circumferential fibers shorten simultaneously with the oblique fibers in the right- and left-handed helices to thicken the myocardium and empty the heart. We have found that MWF was associated with a selective reduction in circumferential strain, suggesting that MWF preferentially affects mid-myocardial, circumferential fibres. As noted by Buckberg [13], circumferential fibers provide a horizontal counterforce, or 'buttress' to the simultaneously contracting oblique fibres. Impaired circumferential contraction would be expected to lead to impaired rotation, as we have found in patients with MHF. Our finding of more frequent rigid LV body rotation supports the notion that MWF renders the LV less capable of twisting and more liable to move as a rigid body.

We have previously shown that patients with NICM and MWF treated with CRT are more likely to suffer pump failure than patients without MWF [10]. On the other hand, Lamia et al. found that CRT improved torsion, stroke volume and stroke work in an animal model [20]. Using 3-dimensional speckle-tracking echocardiography, others found that in patients with NICM, CRT led to an improvement in LV torsion [21]. If torsion is indeed influenced by CRT, we might expect that the higher risk of pump failure observed in patients with MHF undergoing CRT may be due to a permanent inability of the LV to twist and untwist. This hypothesis requires further exploration.

### Diastole

In diastole, release of energy stored in systole (recoil) causes rapid untwisting and a mitral-to-apical negative gradient [22] that 'sucks' blood from the left atrium to the LV [23]. Untwisting occurs mainly during the isovolumic relaxation period and is followed by diastolic filling. Several studies [24–26] have shown that whilst cavity volume is fixed during isovolumic relaxation, there is a rapid recoil of about 40 % of the torsion effected during systole. We have found that MWF leads to both a multi-directional impairment in diastolic strain rate, as well as to impairment of apical untwist rate. This is likely to account for the higher LV filling pressures observed using echocardiography in patients with NICM and MWF [27]. Conceivably, impaired apical untwisting leads to impaired LV suction and to increased LV filling pressures.

### Limitations

The LGE-CMR technique described herein only detects replacement fibrosis. The more recent technique of T1 mapping, which detects interstitial fibrosis, was not undertaken. We cannot therefore comment as to whether our findings are also influenced by the latter. In addition, we have not routinely undertaken myocardial biopsy, nor have we quantified myocardial oedema. Therefore, we cannot exclude the possibility that our findings were influenced by active myocarditis, despite the absence of evidence from clinical and laboratory screening. We should also add that different manufacturers have varying methodologies for the calculation of the mechanical variables described and therefore, our findings are not generalizable to other FT-CMR methodologies. Publication of the FT-CMR algorithms used by different manufacturers would be welcome.

## Conclusions

We have shown that in patients with NICM, MWF is associated with profound disturbances in LV global circumferential strain, strain rate, LV twist and torsion, in both systole and diastole. In addition, MWF is associated with rigid LV body rotation. These findings provide a mechanistic link between MWF and a poor clinical outcome in patients with NICM, despite pharmacologic and device therapy.

## Abbreviations

ACE-I: angiotensin-converting enzyme inhibitors
ARB: angiotensin receptor blockers
CMR: cardiovascular magnetic resonance
DSR: diastolic strain rate
Ɛ: strain
FT: feature-tracking
LGE: late gadolinium enhancement
LV: left ventricle/ventricular
MWF: mid-wall fibrosis
NICM: non-ischemic cardiomyopathy
SSR: systolic strain rate
